# Neoadjuvant therapy-induced remodeling of tumor immune microenvironment in pancreatic ductal adenocarcinoma: a spatial and digital pathology analysis

**DOI:** 10.1007/s00428-025-04056-y

**Published:** 2025-02-27

**Authors:** Danting Li, Yongjun Liu, Ruoxin Lan, Venu G. Pillarisetty, Xiaofei Zhang, Yao-Zhong Liu

**Affiliations:** 1 Department of Biostatistics and Data Science, Celia Scott Weatherhead School of Public Health and Tropical Medicine, Tulane University, New Orleans, LA USA; 2 Department of Laboratory Medicine and Pathology, University of Washington School of Medicine, Seattle, WA USA; 3 Department of Surgery, University of Washington School of Medicine, Seattle, WA USA; 4 Department of Pathology and Laboratory Medicine, University of Wisconsin-Madison, Madison, WI USA; 5 Department of Pathology and Laboratory Medicine, New York University Grossman Long Island School of Medicine, Long Island, NY USA

**Keywords:** Neoadjuvant therapy, Pancreatic cancer, Tumor immune microenvironment, Spatial analysis, Digital pathology

## Abstract

Neoadjuvant therapy (NAT) is the standard of care for borderline-resectable and locally advanced pancreatic ductal adenocarcinoma (PDAC). It can be used to treat resectable PDAC. This study aimed to investigate how NAT remodels the tumor immune microenvironment (TIME) and whether this remodeling translates into survival benefits. We performed spatial and digital pathology analysis of 27 upfront resection patients (naïve group) and 39 age-, gender-, and stage-matched patients who had surgery after NAT (NAT group). AI-assisted digital pathology was used to annotate cancer cells and CD8 + T lymphocytes. Spatial correlation between CD8 + T lymphocytes and cancer cells for each case was assessed using spatial point pattern analysis, followed by generalized linear modeling (GLM) of quadrat counts of CD8 + T cells, with the quadrat counts of cancer cells as the independent variable. The regression coefficient was used to quantify the strength of their spatial correlation and then further assessed for association with patient survival. The analyses showed that the NAT group, compared with the naïve group, had increased spatial correlation of CD8 + T cells with cancer cells, suggesting enhanced effector T cell-cancer cell engagement in the NAT patients. Additionally, patients with a higher degree of spatial correlation between the two cells showed improved after-surgery survival. Through a new methodological framework that takes advantage of AI-assisted digital pathology and spatial point pattern analysis, our study has successfully captured the subtle effects of NAT-induced TIME remodeling and assessed its impact on prognosis of PDAC patients.

**Supplementary Information:**

The online version contains supplementary material available at 10.1007/s00428-025-04056-y.

## Introduction

Pancreatic ductal adenocarcinoma (PDAC) is a highly lethal malignancy, with a 5-year overall survival rate of only 11% [1]. This poor prognosis is mainly attributed to the aggressive nature of the disease, late presentation with advanced symptoms, resistance to current therapies, and challenges in early detection [2]. Neoadjuvant therapy (NAT) is now increasingly used in the management of PDAC, particularly for cases deemed resectable and borderline-resectable [3–5], as the therapy was found to promote the potential of margin-negative resections, to treat micro-metastases early, and to select patients with favorable tumor biology for surgery [5, 6]. Additional advantages of NAT include in vivo assessment of therapeutic response and conversion of locally advanced disease to resectable status [7].

NAT has been shown to induce immune cell infiltration and potentially remodel the tumor immune microenvironment (TIME) in PDAC. A recent study found that NAT increased CD8 + T lymphocyte density and decreased T regulatory cell and M2 macrophage density, with the latter associated with improved survival [8]. Although this study did not find association of CD8 + T lymphocyte density with patient survival, earlier studies highlighted the importance of CD8 + TILs (tumor infiltration lymphocytes) in PDAC prognosis [9]. In particular, a recent meta-analysis found that high infiltration of CD8 + or CD4 + TILs was associated with better overall and disease-free survival [10]. Additionally, a study comparing neoadjuvant chemoradiation (CRT) to no treatment in PDAC patients showed increased CD3 + T cell infiltration and overexpression of genes involved in antigen presentation and inflammation in CRT-treated tumors [11]. A high ratio of Tregs to total T cells was associated with poor survival in CRT-treated patients [11].

The complexity of PDAC TME has been extensively studied [12]. Key players in the PDAC TME cellular component include immune cells (e.g., macrophages, neutrophils, dendritic cells, CD8 + T cells, etc.), endothelial cells, pancreatic stellate cells, cancer-associated fibroblasts, and myofibroblasts. Those in the TME extracellular component include collagen, fibronectin, and soluble factors, such as cytokines, chemokines, and complements. Interactions between these factors play an important role in the dynamics of TME remodeling, which may explain the commonly observed clinical events, such as therapeutic resistance, immunosuppression, and elusion of immune surveillance. Recent advances in spatial transcriptomics/proteomics and spatial point pattern analyses have enabled detailed profiling of these factors, shedding light on their roles in PDAC TME [12].

Not only the density, the spatial relationship between TILs and cancer cells is also crucial for their prognostic significance. Using spatial computational analysis, Masugi et al. [13] found that the CD8 + cell density in the tumor center was associated with patient survival, yet the density in the tumor margin correlated with CD274 (PD-L1) expression and tertiary lymphoid structures. This study highlighted spatial heterogeneity of CD8 + T cell densities in PDAC TME and the importance of region-specific analysis. The cell’s spatial heterogeneity was further confirmed by another study [14] using a computational imaging technology for simultaneous evaluation of eight distinct markers, which found that the abundance of cytotoxic T cells (the CD8 + cells) in proximity to cancer cells of PDAC patients were associated with a better survival and PDAC desmoplasia may not be a physical barrier for T-cell accumulation. Further research was conducted using co-detection by indexing (CODEX) technology to characterize PDAC tissue regions with seven protein markers [15], which identified a distinct pattern of cellular neighborhoods of CD8 + T cells, characterized by the cells being closers to themselves than to the cancer cells, potentially as a manifestation of an immune evasion mechanism. The study also established the pattern’s association with a poor prognosis. Another spatial point pattern analysis of data assayed with a 27-plex marker panel [16] highlighted the role of the relative distance among immune cell populations (e.g., IL10 + myelomonocytes, PD-1 + CD4 + T cells, and granzyme B + CD8 + T cells) in PDAC patient survival. Omics-based mechanistic studies [17–19] further characterized the immune landscape of PDAC TME. These studies revealed several key markers related to patient survival. Specifically, CD3, CD206, CD8, and CD68 were identified as TME markers related to patient survival [17]. Pro-inflammatory chemokines, granzyme B, and alpha-smooth muscle actin + fibroblasts were found to be associated with non-recurrent PDAC [18]. Additionally, higher densities of cytotoxic T lymphocytes (CD8 + T cells) and the upregulation of T-cell priming-associated genes (such as CD40, ITGAM, glucocorticoid-induced TNF-related receptors) were identified as TME markers for high-immunogenic PDAC [19].

These studies have highlighted the crucial role of CD8 + T cells in PDAC prognosis. However, a limitation in prior research is a lack of quantification of local cancer cell density alongside CD8 + T cells, which may provide critical context for TME dynamics. Advances in digital pathology, integrating AI, machine learning, and big data, allow for more precise and automated quantification of cancer and immune cells, transforming oncology research and diagnosis [20–22]. In this study, we tried to address this gap by quantifying both CD8 + T cells and cancer cells using AI-assisted digital pathology and analyzing their spatial correlation to understand immune response dynamics in PDAC and the effects of NAT on TME remodeling. Here, we hypothesize that spatial correlation between CD8 + T cells and PDAC cancer cells contributes to better patient survival and may be enhanced by NAT. We employed AI-assisted digital pathology to annotate, enumerate, and spatially locate cancer cells and CD8 + T cells. The spatial correlation between CD8 + T cells and cancer cells was analyzed using spatial point pattern tools, with its association to patient survival tested via Cox proportional hazards models.

## Materials and methods

### Patient cohort and data collection

The experimental design and protocols for patient selection and data collection were reviewed and approved by the Institutional Review Boards at the University of Wisconson-Madison (UW-Madison). All PDAC patients who underwent surgical resection of PDAC at UW-Madison between 2015 and 2020 were retrospectively reviewed. The inclusion criteria were PDAC patients from both genders with complete clinicopathologic information and complete archived histologic slides and tissue blocks. PDACs arising from intraductal papillary mucinous neoplasm (IPMN) or other cystic lesions were excluded. A total of 66 patients were identified as meeting the study criteria, including 39 patients who had NAT (either chemotherapy alone or in combination with radiation) before surgery, and 27 patients matched by age, gender, and stage and underwent upfront surgery without any forms of NAT. The pertinent clinicopathological information was retrospectively collected by reviewing electronic medical records. These data were de-identified prior to analysis. Among the 39 patients in the NAT group, 24 patients received multiple cycles of FOLFORINOX (including 6 patients also received concurrent radiation therapy), and 15 patients received multiple cycles of gemcitabine and Abraxane (including 6 patients also received concurrent radiation therapy). Fourteen patients received concurrent radiation therapy. For all patients, only resection specimens were used. As the purpose of the study is to investigate the spatial correlation of the cancer and CD8 + T cells at the level of quadrats, cases with inadequate numbers (< 10) of either cancer cells (case IDs: 52, 70, 71, 72) or CD8 + T cells (case ID: 36) due to inadequate areas of residual cancer areas on the slides were excluded from analyses downstream of quadrat count analysis.

### Histology and immunohistochemistry (IHC)

Serial Sects. (4 μm) were cut from the selected formalin-fixed, paraffin-embedded (FFPE) tissue blocks for the consecutive staining of H&E and IHC staining. For IHC, sections were stained using the Roche Ventana Medical System’s Discovery Ultra Automated Platform (Roche Diagnostics, USA). All reagents were Roche-Ventana proprietary reagents except for the Harris Modified hematoxylin (ThermoFisher). Sections from human tonsils were used as positive controls for CD8 + T cells. After deparaffinization, heat-induced epitope retrieval was done with CC1 buffer (Ventana #950–224) for approximately 32 min at 95 °C. Then, slides were incubated with the primary antibody (anti-CD8, Santa Cruz). After reaction with horseradish peroxidase, all slides were counterstained with hematoxylin, and digitalized using the AperioDigital Pathology Scanner at 40 × resolution.

### Digital pathology analysis with Aiforia

**Fig. 1:**
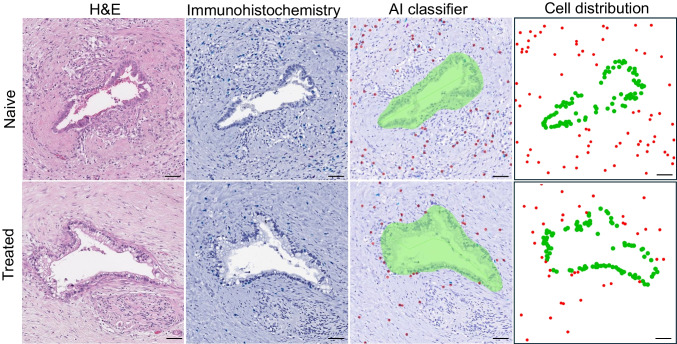
Artificial Intelligence assisted characterization of the TME remodeling by NAT. First row: a naive group patient (case #4). A H&E image (1st column) and a CD8 immunostaining (2nd column) image of a PDAC resected from the naive group. AI-based tissue classifiers were trained using Aiforia to label malignant glands in green, and CD8 + T cells in red (3rd column). The spatial coordinates of AI-recognized CD8 + T cells (red points) and cancer cells (green points) were used to generate a map (4th column) for downstream spatial pattern point analysis. (scale bar: 500 μm). Second row: an NAT treated group patient (case #31). Note the higher density of CD8 + T cells in the close neighborhood of cancer glands in NAT group

Tumor glands in different slides show a spectrum of histomorphologic objects, including well-differentiated tumor glands, small clusters of cancer cells, and singly infiltrating poorly differentiated cancer cells. The whole slide image (WSI) of each slide was meticulously selected to represent a diverse and comprehensive collection, reflecting histological and pathological heterogeneity of PDAC. For the analyses of digitized WSIs of HE and IHC-stained slides, we utilized Aiforia’s cloud-based platform (Aiforia Technologies, Helsinki, Finland), which provides advanced tools for machine learning/deep learning-based digital pathology analytical pipelines and workflows. The algorithm setup involved training the system to recognize and classify various histopathological features (cellular morphology, tissue architecture, nuclear features, stromal components, inflammatory infiltrates, tumor characteristics) important to PDAC. Specifically, key system parameters were trained and tuned to detect malignant PDAC glands and cells, CD8 + lymphocytes, and stromal components within the tissue sections. This was achieved through an initial supervised training of the system for annotation of important features/structures, which was conducted by two experienced GI pathologists (XZ and YJL, who were blind to the treatment group of the cases) through Aiforia Create in the following steps. Figure 1 shows two cases (a naïve patient, case #4 vs. an NAT patient, case#31) of AI-assisted characterization of the TME.

First, tumor areas were selected in a WSI, where multiple representative regions of interest (ROIs) in the tumor areas were chosen. Then, meticulous annotations of the ROIs were performed to identify typical tumor glands and cancer cells, CD8 + lymphocytes, and desmoplastic stroma. A consensus was achieved for all the annotations between the two pathologists. Such pathologist-supervised annotation procedure was performed for each case. The CNN (convolutional neural networks) algorithm enabled by Aiforia was then trained based on the annotated features, and the trained algorithm was then applied to the whole field of the WSI. The algorithm’s performance was iteratively improved by the two pathologists’ thorough evaluation of the system-annotated images, followed by adding more human-annotated examples (as training examples) if the machine-delivered annotation results were not satisfactory. By doing so, training parameters were fine-tuned by incorporating feedback from the two pathologists. This process was repeated multiple times to enhance the algorithm’s accuracy and robustness until satisfactory results were achieved. For the supervised training of the Aiforia system by the two pathologists, more detailed steps are illustrated below.

Step 1: Upon a thorough and careful examination of the whole scanned slide, the two pathologists (XFZ and YJL) reached a consensus in selecting and manually annotating at least 5 well-defined, representative regions of typical PDAC tumor glands or infiltrating tumor cells, which were used for initial training of deep learning (DL)-based tumor classifier. In this selection process, a distinction of reactive non-neoplastic atypical pancreatitis glands from PDAC tumor glands was achieved by the consensus of the two pathologists to ensure that the selected regions did not contain non-neoplastic glands. Step 2: Within each of the selected regions, a few CD8 + T cells and tumor gland cells were chosen by the pathologists as examples to train Aiforia to learn the key morphological features of the targets and then start the process of recognizing, annotating, and enumerating the target cells using the DL algorithm. Step 3: The DL-based classifiers for tumor and CD8 + T cells were then tested in a formal analytical field (a much larger tumor region encapsuling the initial training regions, their surrounding areas and beyond) for multiple rounds of quality improvement. In this step, the two pathologists independently and double blindly conducted a thorough evaluation of the performance accuracy of Aiforia. Specifically, focusing on a manageable number of cells, two performance metrics, PPV (positive predictive value), i.e., proportion of true positives among all Aiforia positively labeled cells, and NPV (negative predictive value), i.e., proportion of true negatives among all cells not labeled by Aiforia, were assessed based on the pathologists’ judgement on the target cells as the gold standard. If PPV or NPV < 90%, then Step 2 was repeated by the two pathologists, where they focused on manually annotating those false positives and false negatives for further training, following by re-assessment of the new annotation results by the improved DL-based classifiers (Step 3). This iteration process of re-training and re-assessment was normally repeated for three to five times until the PPV/NPV values reached > 90%. Only at this point the analytical results, i.e., cell annotation and *x*–*y* positions of the annotated cells, were finalized and exported for downstream analyses.

### Spatial point pattern analysis

**Fig. 2:**
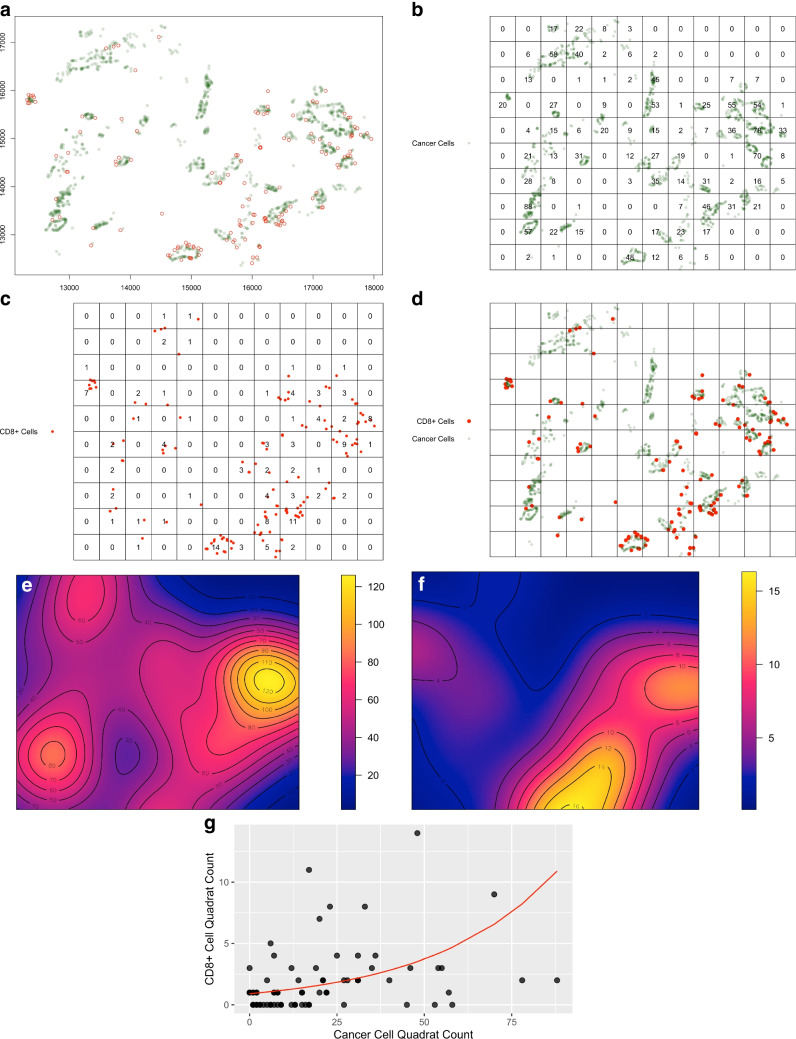
Quadrat count analysis for an NAT-treated patient (case #1) as an example. **a** The *X*–*Y* coordinate map for CD8 + TILs (red) and cancer cells (green) from raw data collected with the Aiforia system. **b**–**d** The raster-based maps with quadrat counts for cancer and CD8 + T cells, respectively, created with the spatstat software. In **d**, the maps shown in **b** and **c** were overlayed to highlight the spatial correlation of two types of cells. **e**, **f** The kernel density maps for cancer and CD8 + T cells, respectively, created with the spatstat software based on the raster-based maps. **e** and **f** were created to illustrate “smoothed” density maps, which were inferred from the raw cell counts as observed in **b** and **c**, respectively, based on a quartic kernel function. In the maps, the scale of cell density (cell counts in mm^2^) was shown with a continuum of color spectrum, where the warmer color (more yellowish) indicates a higher density. The color scales with the corresponding cell densities are shown in the color spectrum band to the right. The black contour lines within the map indicate lines of equal cell densities and illustrate the overall distribution and variation of cell densities across a whole region. **g** Scatter plot and the fitted regression line based on generalized linear modeling for quadrat counts of the two cells. Quadrat counts of two types of cells were paired by the row-column location, e.g., the cancer cell count of 20 in row 4 and column 1 in B was paired with the CD8 + T cell count of 7 in row4 and column 1 in C. For a quadrat with a pair of counts being both 0, that quadrat was excluded from downstream analysis

For every annotated object (cancer cells or CD8 + lymphocytes), the location (i.e., *X*–*Y* coordinate) information within an Aiforia-annotated image file was generated by Aiforia, by which a distance measure (i.e., the shortest distance in $\mu$ m) from every CD8 + T cell to tumor gland was also generated. In order to selectively study only tumor infiltrating lymphocytes (TILs) which are geographically close to cancer and believed to interact with cancer cells for immune surveillance, we filter and select only CD8 + T cells with a distance less than or equal to 50 $\upmu$ m to tumor gland for downstream analyses. A scatterplot was generated with R, showing the distribution of CD8 + T cells within cancer cell population (Fig. 2a from case #1 as an example).

Spatial point pattern analysis was performed using the spatstat R package [23]. Specifically, the xlsx file was read and split into 2 separate dataframe R objects, containing information for only cancer cells or CD8 + cells, respectively. Then, the dataframe objects were converted into spatstat “ppp” objects using the “ppp” function of the package. A 1:1000 rescaling was performed to convert the *x*–*y* coordinate unit from $\upmu$ m to mm using the “rescale” function. Using the ppp object, kernel smoothed intensity for the spatial distribution of cancer or CD8 + T cells was computed with the “density” function, which was then plotted with intensity contour lines using “plot” and “contour” functions (Fig. 2e, f as examples).

Using the created ppp objects for either cancer cells or CD8 + T cells, grids of quadrats were set up with a unit quadrat size of 500 um × 500 um so that the number of cells can be counted within each quadrat (Fig. 2b–d as examples). To assess the spatial correlation between the two cells within a patient’s sample, quadrat counts of CD8 + T cells were modeled in a generalized linear model (GLM) as the dependent variable (following a negative binomial distribution), with the quadrat counts of cancer cells as the explanatory variable. Those quadrats with a count of 0 for both cells were removed from analysis. As an example, Fig. 2g shows the scatterplot with *x*-axis showing the cancer cell quadrat count and *y*-axis showing the CD8 + cell quadrat count. The red line is the fitted curve from the GLM modeling. The statistical modeling of GLM was performed using the glm.nb function rendered by the MASS R package [24].

### Other statistical analyses

The glmmTMB package [25] was used for generalized linear mixed modeling of CD8 + T cell count, with cancer cell count and treatment group as explanatory variables. The survival package [26] was used for KM analysis and Cox proportional hazards model analysis. The package survminer was used to construct the KM plot with risk table (Fig. 5) and the hazard ratio forest plot (Fig. 6). Several other R packages, including ggplot2 [27], gridExtra, and svglite packages, were also used to generate other plots and graphs in this work.

## Results

### Clinicopathological characteristics of patients

There are a total of 66 patients (30 females and 36 males) involved in the study, with age ranging from 46 to 88, among whom, 27 patients underwent naïve treatment and 39 NAT treatment. Table 1 presents detailed characteristics in all the subjects as a whole and in separate groups of NAT and Naïve patients. A supplemental table is also provided for additional clinicopathological data, including lymphovascular invasion (LVI), perineural invasion (PNI), margin status (the presence of cancer cell at the margin of surgical resection), and lymph node status ( i.e., how many nodes are positive for metastatic cancer among all lymph nodes harvested from the specimen).

**Table 1:** Patient characteristics

	All	Naïve	NAT	*p* value
Total (*n*)	66	27	39	
Median age (range)	70 (46–88)	70 (54–87)	69 (46–88)	0.83
Age group *n* (%)				
≤ 70	34 (51.5)	14 (51.9)	20 (51.3)	0.96
> 70	32 (48.5)	13 (48.1)	19 (48.7)	
Gender, *n* (%)				
Male	36 (54.5)	19 (70.4)	17 (43.6)	0.03
Female	30 (45.5)	8 (29.6)	22 (56.4)	
Tumor size	2.9 (0.5–7)	3.5 (1.5–7)	2.5 (0.5–4.5)	0.00038
T stage				
T1	11 (16.7)	1 (3.7)	10 (25.6)	0.0007
T2	22 (33.3)	5 (18.5)	17 (43.6)	
T3	33 (50)	21 (77.8)	12 (30.8)	
T4	0	0 (0)	0	
N stage				
N0	24 (36.4)	7 (25.9)	17 (43.6)	0.338
N1	36 (54.5)	17 (63.0)	19 (48.7)	
N2	6 (9.1)	3 (11.1)	3 (7.7)	
TNM stage				
I	16 (24.2)	4 (14.8)	12 (30.8)	0.316
II	38 (57.6)	19 (70.4)	19 (48.7)	
III	10 (15.2)	3 (11.1)	7 (17.9)	
IV	2 (3.0)	1 (3.7)	1 (2.6)	
Grading				
G1	12 (18.2)	6 (22.2)	6 (15.4)	0.735
G2	33 (50)	13 (48.2)	20 (51.3)	
G3	21 (31.8)	8 (29.6)	13 (33.3)	
TRG (CAP)				
0			0	
1			1	
2			20	
3			18	
Event (death) observed/censored	48/18	19/8	29/10	
After surgery survival time (death observed) (days)	Range: 90–1769 Mean: 569 Median: 457	Range: 134–1496 Mean: 719 Median: 706	Range: 90–1769 Mean: 471 Median: 369	
After surgery follow-up time (censored) (days)	Range: 538–2691 Mean: 1539 Median: 1522	Range: 1259–2691 Mean: 2102 Median: 2206	Range: 538–1749 Mean: 1090 Median: 916	

### Quadrat count analysis

In order to minimize the impact of intratumoral heterogeneity, we first annotated the entire tumor area in each section and then trained AI-assisted tissue classifier to identify and locate (i.e., obtain the *x*- and *y*-coordinates) of all cancer cells and CD8 + T cells in the tumor area (shown in Fig. 1 as an example). Comparison with the manual annotation by two GI pathologists was done for each case as validation. Then, quadrat count analysis was performed on each sample, where number of annotated cancer cells and CD8 + T cells inside each quadrat was assessed by the spatstat package [23]. As an example, Fig. 2 shows quadrat count data and the associated raw data and analytical results for case #1. Those quadrats (mostly outside of the cancer region) with a count of 0 for both cancer and CD8 + cells were excluded from downstream analyses.

Among all 66 patients, the mean total count of cancer cells was 8290 (± SE 1207) and the mean total count of CD8 + cells was 1376 (± SE 258). For each patient’s sample, the mean (quadrat-wise mean) and standard deviation (quadrat-wise standard deviation) of the two cells’ counts across all the quadrats were calculated. The across-patient average for the quadrat-wise mean of the cancer cell counts was 28 (± SE 3). The same measure for the CD8 + cell counts was 5 (± SE 0.4). The across-patient average for the quadrat-wise standard deviation of the cancer cell counts was 26 (± SE 2). The same measure for the CD8 + cell counts was 5 (± SE 0.3).

We compared quadrat-wise standard deviation for cancer cell counts between NAT vs. naïve patients, which was lower in the NAT vs. the naïve groups (Supplementary Fig. 1). The difference between the two groups was statistically significant (*p* = 0.003) after adjustment for sex, age, tumor grade and tumor stage using linear regression modeling. According to the model, on average, the NAT-treated group was 13.34 lower in quadrat-wise standard deviation for cancer cell counts than the naïve patients. This decreased standard deviation of cancer cell quadrat counts in NAT-treated patients may suggest that NAT may generally decrease intratumoral heterogeneity.

### Spatial correlation of CD8 + T cells and cancer cells

We assessed spatial correlation between CD8 + and cancer cells at each subject level. Essentially, for each subject, negative binomial generalized linear model (GLM) analysis was performed across all quadrats with quadrat-count of CD8 + T cells as the dependent variable and the quadrat-count of cancer cells as the independent variable. In the analysis so performed, the regression coefficient (slope, $\beta$) represents the spatial correlation between the CD8 + T and cancer cells in each subject, i.e., the variation of CD8 + T cell density due to per unit number of increase of cancer cell density. Specifically, due to the log link function in the negative binomial GLM model, the coefficient for the regression model predicts an expected “fold change” of *y* (CD8 + cell count) per unit increase of *x* (cancer cell count), e.g., an expected fold change of $\mathrm{exp}\left(n\times \beta \right)$ in CD8 + T cell count per $n$ cancer cells count increase. For example, patient 1 (an NAT-treated subject) achieved a $\beta$ value of 0.0282, which translates into an expected 1.33 (= $\mathrm{exp}\left(10\times 0.0282\right))$fold change (i.e., a 33% increase) of CD8 + T cells with an increase of 10 cancer cell count. Among all the naïve patients, the median coefficient ($\beta$) achieved in the GLM was 0.0078, which translates into an expected fold change of 1.08 of CD8 + T cells (i.e., an 8% increase) associated with an increase of 10 cancer cell count. In contrast, among all the NAT-treated patients, the median coefficient ($\beta$) achieved in the GLM modeling was 0.017, which translates into an expected fold change of 1.19 of CD8 + T cells (i.e., a 19% increase) associated with an increase of 10 cancer cell count.

**Fig. 3:**
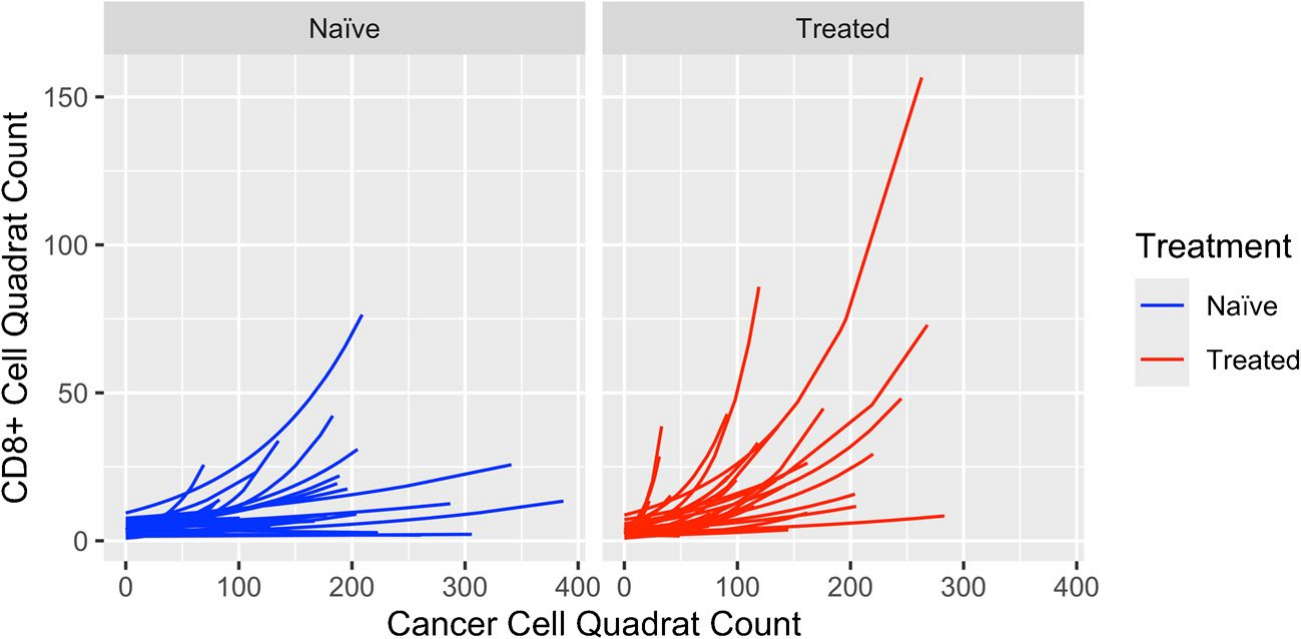
Fitted regression lines achieved in GLM modeling of CD8 + T cell and cancer cell quadrat counts

**Fig. 4:**
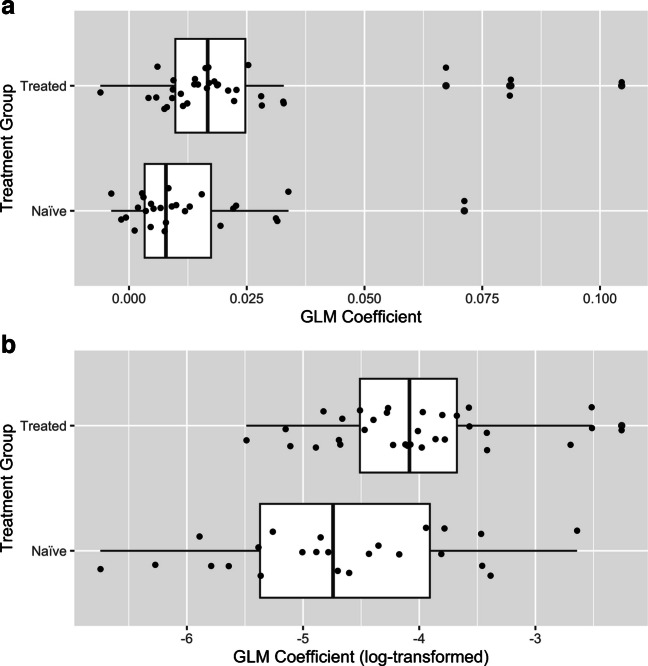
Boxplots comparing slope coefficients achieved in GLM modeling of CD8 + T and cancer cell quadrat counts between NAT-treated and naïve patients. **a** GLM coefficients were presented in original scale. **b** GLM coefficients were presented in log-transformed scale

Here, the scatter plots for CD8 + T cell vs. cancer cell count data and the fitted models (shown in red regression lines) for all the 61 patients were presented in Supplemental Fig. 2 (in four subsets). The fitted regression lines were also presented and compared at the treatment group level in Fig. 3. Using boxplots, the slope coefficients (in original scale as well as in log scale) were presented and compared between the two groups in Fig. 4. From Figs. 3 and 4, it appears that NAT-treated patients had a higher spatial correlation than the naïve patients, as evidenced by a steeper trend of increase of CD8 + T cells associated with the increase of cancer cells at quadrat level (Fig. 3) and higher coefficients (achieved in the GLM modeling) at the subject level, as shown in the boxplots (Fig. 4).

### Difference of spatial correlation of the two cell types between NAT and naïve groups

We then tested the statistical significance for the treatment group difference of spatial correlation in the following analyses. We hypothesized that the slope was higher in the treated than in the naïve groups, as indicated in Figs. 3 and 4. Using linear regression analysis, we modeled the slope coefficient as the dependent variable, with sex, age, treatment (NAT vs. naïve), tumor grade, and tumor stage as independent variables. Due to the right skewness of the distribution of the slope coefficient and hence the obvious deviation from normal distribution (see Fig. 4a), the slope coefficient was log-transformed for normalization (Fig. 4b). The linear regression confirmed the statistical significance for the difference of GLM regression coefficient between the two groups, with a *p* value of 0.0064. The difference of GLM regression coefficients between the two groups at the log-scale is 0.72, which translates into an expected ratio of 2.05 for the GLM coefficients for the NAT over the naïve group.

We further compared the spatial correlation of CD8 + T cells and cancer cells in NAT vs. naïve patients using a more sophisticated approach, the generalized linear mixed model (GLMM) that modeled the CD8 + T cell quadrat count under negative binomial distribution. Specifically, we used the glmmTMB package [25] to model CD8 + T cell count as the dependent variable, and the cancer cell count, treatment (NAT vs. naïve), and the interaction between the two factors as explanatory variables for fixed effects, while adjusting for variations/correlations between subjects as the random effects. The regression coefficient for fixed effects from “cancer cell count” was 0.0047 and achieved a significant *p* value of 2e − 16. The regression coefficient for fixed effects from “treatment (NAT vs. naïve)” was non-significant with a *p* value of 0.477. The regression coefficient for fixed effects from the “interaction between cancer cell count and treatment” was 0.0022 and achieved a significant *p* value of 4.22e − 10.

The strong significance of this interaction term indicated a significantly higher spatial correlation between CD8 + T cells and cancer cells in the NAT vs. the naïve patients. Specifically, based on the model’s estimated parameters, for the naïve patients, it is expected that for each increase of 100 cancer cells, the fold change of CD8 + cells will be 1.60 (= exp(0.0047 * 100)), i.e., an increase of 60% at the quadrat level. For the NAT-treated patients, it is expected that for each increase of 100 cancer cells, the fold change of CD8 + T cells will be 1.99 (= exp((0.0047 + 0.0022) * 100)), i.e., an almost one-fold increase at the quadrat level. Importantly, comparing the above two expected outcomes, it was shown that for each increase of 100 cancer cells, the fold change of CD8 + T cells at quadrat level is significantly higher (*p* = 4.22e − 10) in the NAT than the naïve patients, with the ratio of the fold changes being 1.25 (= exp(0.0022 * 100)), i.e., the fold change is 25% higher in NAT than naïve patients.

### The impact of spatial correlation between CD8 + T cells and cancer cells to patient survival

According to Kaplan–Meier (KM) analysis, the median survival time for the 61 subjects (i.e., the total 61 patients after excluding the five patients with case IDs of 36, 52, 70, 71, 72) was 678 days, with 95% confidence intervals as 439–1125 days.

**Fig. 5:**
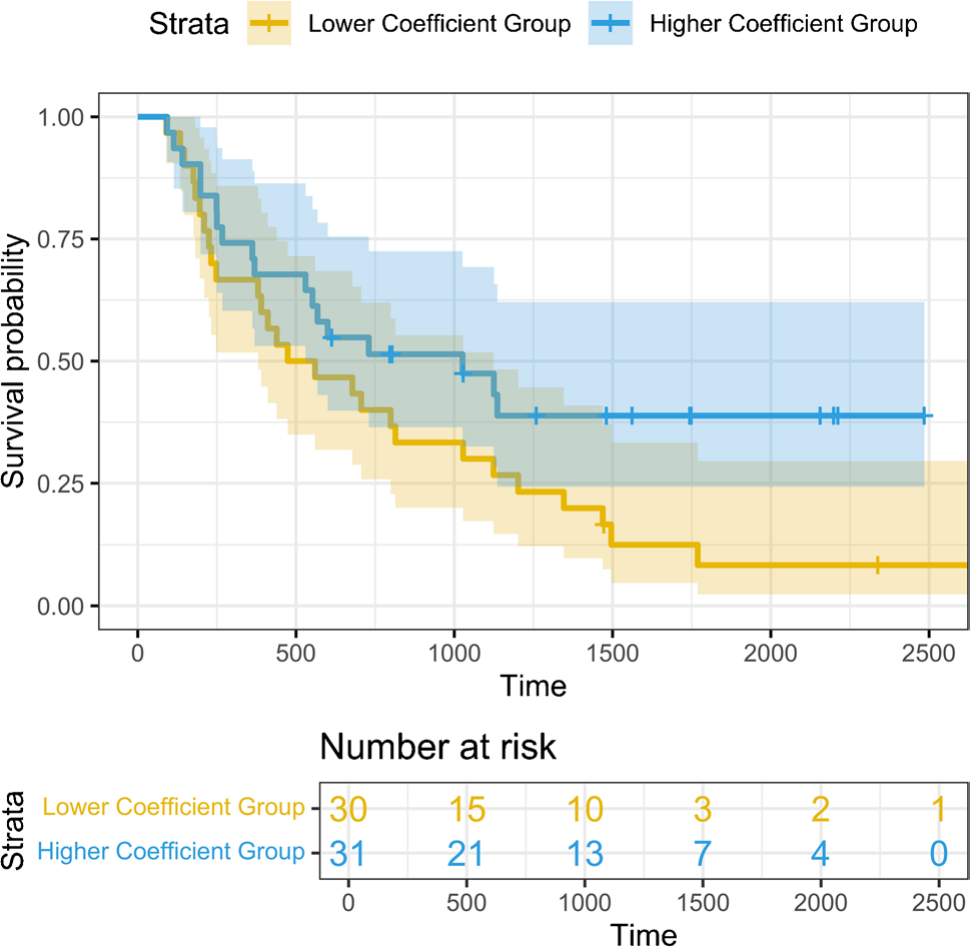
Kaplan–Meier curve comparison for patients with higher vs. lower slope coefficients from spatial correlation analysis of CD8 + T and cancer cells

We used the slope coefficient estimates obtained in the GLM analysis of CD8 + T cells quadrat counts against cancer cells quadrat counts (see Figs. 3 and 4 and Supplemental Fig. 2) to represent the strength of the spatial correlation of the two cells for each patient. The estimated slope coefficient values ranged from − 0.006 to 0.105, with the median as 0.013. Based on the median of the slope values, we separated the patients into the “Higher Slope Coefficient vs. Lower Slope Coefficient” groups, with those patients whose coefficient ≥ median as the former and the remaining patients as the latter group. The KM survival curves for the two groups of patients are shown in Fig. 5, which indicated a better survival for the Higher Slope Coefficient group.

**Fig. 6:**
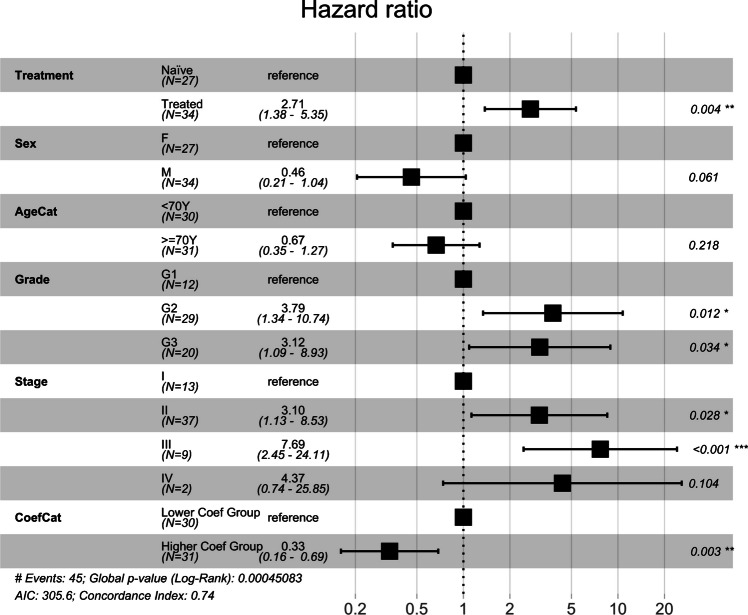
Hazard ratio forest plot based on multivariate Cox proportional hazards model analysis of all patients

We further tested the statistical significance of the slope coefficient grouping for predicting patient survival using a multivariate Cox proportional hazards model, which was constructed with predictors including treatment, sex, age, tumor grade, tumor stage, together with the group assignment based on the slope coefficients achieved in the GLM analysis involving the two cells’ quadrat counts. To better illustrate the hazard ratio for age, a new categorical variable, AgeCat, was created based on the median of age (70 years) as the cut-off, with age ≥ 70 as the upper level and age < 70 as the lower level (the reference level). As shown in the result (Fig. 6), after adjustment for all the covariates, the Higher Slope Coefficient group (patients with a stronger spatial correlation between the two cells at the quadrat level) was shown as a significant predictor for better survival (*p* = 0.003), as compared to the Lower Slope Coefficient group (patients with a weaker spatial correlation between the two cell types at the quadrat level), with a hazard ratio (HR) as 0.33 and the 95% Confidence Interval (CI) as 0.16 to 0.69. As expected, higher tumor grades (G2 and G3) and tumor stage (stages II and III) were also significant predictors for worse survival (as compared to the baseline grade and stage). Lastly, the treatment NAT was shown to be a negative predictor for survival (*p* = 0.004).

## Discussion

In this work, we used the state-of-the-art AI-assisted digital pathology technology to recognize and quantify PDAC cancer cells and CD8 + TILs in tissue histology slides from 66 patients, who were either treated with NAT before surgery (the NAT group) or underwent upfront surgery without NAT (the naïve group). We then examined the spatial distribution and correlation of the two cell types using spatial point pattern analysis. As the major aim of the research, we focused on the spatial correlation between the two types of cells as a key measure for evaluating the degree of CD8 + T cell-cancer cell engagement, followed by assessing the impact of NAT on the spatial correlation of the two cells. We hypothesized that NAT may lead to a stronger spatial correlation between CD8 + T and cancer cells, thereby enhancing T cell-cancer cell engagement. We also hypothesized that a stronger spatial correlation, which may reflect more effective immune surveillance by CD8 + T cells, could contribute to improved survival outcomes for PDAC patients.

Our results provided supportive evidence for the above hypotheses. These include higher slope coefficients in NAT vs. naïve patients for association between CD8 + T cell and cancer cell quadrat counts (Figs. 3 and 4, Supplemental Fig. 2). Through a more advanced model, the GLMM, the above finding was confirmed by an observation of a “higher degree of increase” of CD8 + T cells in NAT vs. naïve patients in response to the same number of cancer cell increase. In addition, patients with a stronger spatial correlation for the two cell types, defined as those with the slope coefficients in the upper 50th percentile, were shown to have a better survival outcome.

Our results are consistent with previous studies highlighting the prognostic importance of CD8 + T cells as a key component of PDAC TME and as an active responder involved in TME remodeling induced by NAT. Previous studies have shown that NAT may increase CD8 + T cell density of PDAC patients [8], and this finding, together with the evidence of better survival associated with an increase of densities of CD8 + T cells [9, 10], suggested a beneficial treatment effect of NAT via CD8 + T cell enrichment in PDAC TME. While our findings are in general consistent with these previous studies [8–10], there is a subtle difference. In our study, the densities of CD8 + T cells were normalized by the densities of surrounding cancer cells through quadrat count and spatial correlation modeling, generating slope coefficients that were used for survival analysis. These “normalized” densities of CD8 + T cells may represent a more accurate and robust measure of TILs, as they account for the background densities of cancer cells in the TME, which plays a critical role in modulating the immune response.

Recent studies have characterized CD8 + T cell population in PDAC TME from other perspectives. For example, a region-specific prognostic impact has been emphasized, with one study showing that the density of CD8 + T cells in the tumor center (rather than at the tumor margin) is crucial for survival [13]. In addition, there has been some controversy regarding the prognostic value of CD8 + T cell density near cancer cells. One study [13] found no association, while another [14] reported positive prognostic effect. Our findings align more closely with the latter, as we focused on CD8 + T cells “proximate” to tumor glands, specifically those within 50 $\upmu$ m of the tumor gland. In contrast, there appeared to be a much weaker spatial correlation between CD8 + T cells and cancer cells if all the observed CD8 + T cells (including those cells distant from the tumor glands) were involved in the analysis of our study. The finding from another study [15] offered novel insights into immune evasion of PDAC through spatial analysis of intercellular distances. They found that cases where CD8 + T cells were closer to each other than to the tumor had a poor prognosis. However, we are unable to directly compare our results with theirs as the design of our study did not involve measurement of intercellular distances.

Our study did not include precursor lesions of PDAC, such as pancreatic intraepithelial neoplasia (PanIN), intraductal papillary mucinous neoplasm (IPMN), and intraductal oncocytic papillary neoplasm (IOPN). Hence, a comparison of our results with studies on these precursor lesions [28–30] may not be feasible. These studies have highlighted differences in the TIME between IPMNs and PanINs, particularly for CD8 + T cells, CD4 + T cells, and fibroblasts [28, 30]. While PDACs preceded by PanINs show a loss of CD8 + T cells early on, IPMNs have a higher proportion of CD8 + T cells in low-grade lesions, with a decrease of CD8 + T cells as the lesion progresses to high-grade [28, 30]. IPMNs retain activated CD4 + T cells in various stages of dysplasia. But the cell’s spatial distribution changes with advancing dysplasia [28, 30]. Additionally, PanINs feature restraining fibroblasts, and IPMNs are characterized by cancer-promoting myofibroblasts [28, 30]. A recent study also found that, unlike IPMNs, the progression of IOPN to invasive carcinoma is associated with an increase in CD8 + T cells, suggesting active immune surveillance and potentially explaining the favorable survival rates in IOPN patients [29].

An interesting observation from the survival analysis in our dataset was that the NAT patients had worse survival than the naïve patients. As shown in the Table 1, there was no significant difference in N stage distribution between Naïve and NAT groups (*p* = 0.338). The difference in TNM stage and grading distributions was also statistically non-significant (*p* = 0.316 and *p* = 0.735, respectively). The seemingly contradictory findings, including [1] the survival benefit of an increased spatial correlation between CD8 + T cells and cancer cells, [2] spatial correlation was higher in NAT than the Naïve patients, and [3] yet NAT patients still had poor survival than the Naïve patients, may be explained as follows. Multiple mechanisms may contribute to the survival benefits from NAT to PDAC patients. One key mechanism identified in our study is the increase of spatial correlation of CD8 + T cells and cancer cells. In addition to this, NAT may down-stage the tumor and decrease the tumor size, as evidenced by the significantly lower stages and smaller tumor sizes observed in the NAT patients (Table 1). Despite these benefits, NAT patients still showed worse survival than the Naïve patients in our study, which is likely due to their worse general condition at diagnosis. This baseline condition is not fully captured by Table 1. For instance, tumor size and stage data in Table 1 for the NAT group were collected “after” NAT treatment, which in a more rigorous sense should not be considered “baseline” information. If these data were collected before NAT treatment, the TNM stage of NAT patients may have been worse than, rather than comparable to, that of the naïve group. Therefore, the worse survival for those NAT patients may be largely due to their inferior baseline conditions prior to NAT. This is a typical “selection bias” problem that often occurs in observational clinical studies, which can be mitigated through a formal double-blinded randomized clinical trial (RCT). Unfortunately, our study did not implement an RCT design, which may have led to a contradictory observation of a worse survival in NAT patients despite the association of NAT with a favorable factor (i.e., an increased spatial correlation between CD8 + T cells and cancer cells). To note, although still controversial, some clinical trials supported that NAT may offer survival benefit to PDAC patients [31, 32]. Hence, we may reasonably expect better survival of NAT patients (via NAT’s effects on spatial correlation of CD8 + T cells and cancer cells) should a completely randomized assignment of patients to NAT vs. naïve groups be implemented. More well-designed and rigorously performed RCTs are needed to validate this hypothesis.

In summary, by using a digital pathology approach combining spatial point pattern analysis and AI-assisted annotation, our study quantitatively evaluated spatial correlation between CD8 + TILs and PDAC cancer cells, which may be used as an indicator for CD8 + T cell immune surveillance within PDAC TME/TIME. Our findings are largely congruent with previous studies on the positive effects of CD8 + TILs on patient survival [13–16] and on NAT’s effects in increasing CD8 + TILs [8]. As a key contribution and relevance to field, our study is among the first to demonstrate that NAT may benefit PDAC patients via increasing spatial correlation between CD8 + TILs and cancer cells and highlighted that mechanism as a potential pathway underlying NAT’s favorable impact. We also contributed to the current strategy of PDAC TME analysis by demonstrating the importance and usefulness of quantitating cancer cell density, which has been largely ignored when analyzing CD8 + T cell or other immune cells for immune surveillance. Additional advantages of our study include AI-driven digital pathology techniques that are largely free from human errors and biases in cell annotation and counting process, analytical pipelines that are easy to follow in routine clinical pathology practices and research, and quantitative nature of the findings (i.e., spatial correlation of CD8 + T cells and cancer cells) that are convenient to be developed into a guideline or decision-maker for prognosis and follow-up strategies for PDAC patients. In addition to a lack of randomized clinical trial design, other limitations of our study include a small sample size, lack of replication cohort, and disregard of PDAC precursor lesions. As another key limitation, our study was based on two dimensional snapshots at one point in time and hence the findings may be a simplification of a complicated dynamic process of immune remodeling with TME. To capture the dynamic process, it is ideal that a longitudinal or real-time characterization of immune remodeling is performed based on alternative experimental designs, such as ex vivo cancer slice cultures. Follow-up studies are warranted to address these limitations and further validate our findings.

## Supplementary Information

Below is the link to the electronic supplementary material.

ESM 1 (PNG 39.8 KB)

Supplementary file1 (TIFF 37505 KB)

ESM 2 (PNG 534 KB)

Supplementary file2 (TIFF 337530 KB)

ESM 3 (PNG 562 KB)

Supplementary file3 (TIFF 337530 KB)

ESM 4 (PNG 519 KB)

Supplementary file4 (TIFF 337530 KB)

ESM 5 (PNG 376 KB)

Supplementary file5 (TIFF 337530 KB)

Supplementary file6 (XLSX 12 KB)

## Data Availability

Whole slide scanned images for all H&E slides and immunohistochemistry slides are available from the corresponding authors. Relevant clinical information is included as tables of this manuscript. R codes for spatial analysis and statistics analysis are provided as a supplemental file.

## Declarations

Ethics approval and consent to participate.

The protocols for this study, including slides and tissue block collection and retrieval of all pertinent clinicopathological information from electric medical record, were approved by University of Wisconsin-Madison Institutional Review Boards. This is a retrospective study. Patient consent form was not needed.

## References

[1] Siegel RL, Miller KD, Fuchs HE, Jemal A (2022) Cancer statistics, 2022. CA Cancer J Clin. 72(1):7–33. 10.3322/caac.21708 35020204 10.3322/caac.21708

[2] Kane S, Engelhart A, Guadagno J, Jones A, Usoro I, Brutcher E (2020) Pancreatic ductal adenocarcinoma: characteristics of tumor microenvironment and barriers to treatment. J Adv Pract Oncol. 11(7):693–8. 10.6004/jadpro.2020.11.7.4 33575066 10.6004/jadpro.2020.11.7.4 PMC7646635

[3] Ei S, Takahashi S, Ogasawara T, Mashiko T, Masuoka Y, Nakagohri T (2023) Neoadjuvant and adjuvant treatments for resectable and borderline resectable pancreatic ductal adenocarcinoma: the current status of pancreatic ductal adenocarcinoma treatment in Japan. Gut Liver. 17(5):698–710. 10.5009/gnl220311 36843421 10.5009/gnl220311 PMC10502496

[4] Chawla A, Molina G, Pak LM, Rosenthal M, Mancias JD, Clancy TE, Wolpin BM, Wang J (2020) Neoadjuvant therapy is associated with improved survival in borderline-resectable pancreatic cancer. Ann Surg Oncol. 27(4):1191–200. 10.1245/s10434-019-08087-z 31802297 10.1245/s10434-019-08087-z

[5] Brown ZJ, Shannon AH, Cloyd JM (2024) Neoadjuvant therapy for localized pancreatic ductal adenocarcinoma. Minerva Surg. 79(3):315–25. 10.23736/S2724-5691.23.10150-X 38385797 10.23736/S2724-5691.23.10150-X

[6] Gray S, de Liguori Carino N, Radhakrishna G, Lamarca A, Hubner RA, Valle JW, McNamara MG (2022) Clinical challenges associated with utility of neoadjuvant treatment in patients with pancreatic ductal adenocarcinoma. Eur J Surg Oncol. 48(6):1198–208. 10.1016/j.ejso.2022.02.014 35264307 10.1016/j.ejso.2022.02.014

[7] Silvestris N, Longo V, Cellini F, Reni M, Bittoni A, Cataldo I, Partelli S, Falconi M, Scarpa A, Brunetti O, Lorusso V, Santini D, Morganti A, Valentini V, Cascinu S (2016) Neoadjuvant multimodal treatment of pancreatic ductal adenocarcinoma. Crit Rev Oncol Hematol. 98:309–24. 10.1016/j.critrevonc.2015.11.016 26653573 10.1016/j.critrevonc.2015.11.016

[8] Michelakos T, Cai L, Villani V, Sabbatino F, Kontos F, Fernandez-Del Castillo C, Yamada T, Neyaz A, Taylor MS, Deshpande V, Kurokawa T, Ting DT, Qadan M, Weekes CD, Allen JN, Clark JW, Hong TS, Ryan DP, Wo JY, Warshaw AL, Lillemoe KD, Ferrone S, Ferrone CR (2021) Tumor microenvironment immune response in pancreatic ductal adenocarcinoma patients treated with neoadjuvant therapy. J Natl Cancer Inst. 113(2):182–91. 10.1093/jnci/djaa073 32497200 10.1093/jnci/djaa073 PMC7850539

[9] Miksch RC, Schoenberg MB, Weniger M, Bosch F, Ormanns S, Mayer B, Werner J, Bazhin AV, D’Haese JG (2019) Prognostic impact of tumor-infiltrating lymphocytes and neutrophils on survival of patients with upfront resection of pancreatic cancer. Cancers (Basel) 11(1). 10.3390/cancers11010039 10.3390/cancers11010039 PMC6356339 30609853

[10] Orhan A, Vogelsang RP, Andersen MB, Madsen MT, Holmich ER, Raskov H, Gogenur I (2020) The prognostic value of tumour-infiltrating lymphocytes in pancreatic cancer: a systematic review and meta-analysis. Eur J Cancer. 132:71–84. 10.1016/j.ejca.2020.03.013 32334338 10.1016/j.ejca.2020.03.013

[11] Gartrell RD, Enzler T, Kim PS, Fullerton BT, Fazlollahi L, Chen AX, Minns HE, Perni S, Weisberg SP, Rizk EM, Wang S, Oh EJ, Guo XV, Chiuzan C, Manji GA, Bates SE, Chabot J, Schrope B, Kluger M, Emond J, Rabadan R, Farber D, Remotti HE, Horowitz DP, Saenger YM (2022) Neoadjuvant chemoradiation alters the immune microenvironment in pancreatic ductal adenocarcinoma. Oncoimmunology. 11(1):2066767. 10.1080/2162402X.2022.2066767 35558160 10.1080/2162402X.2022.2066767 PMC9090285

[12] Joseph AM, Al Aiyan A, Al-Ramadi B, Singh SK, Kishore U (2024) Innate and adaptive immune-directed tumour microenvironment in pancreatic ductal adenocarcinoma. Front Immunol. 15:1323198. 10.3389/fimmu.2024.1323198 38384463 10.3389/fimmu.2024.1323198 PMC10879611

[13] Masugi Y, Abe T, Ueno A, Fujii-Nishimura Y, Ojima H, Endo Y, Fujita Y, Kitago M, Shinoda M, Kitagawa Y, Sakamoto M (2019) Characterization of spatial distribution of tumor-infiltrating CD8(+) T cells refines their prognostic utility for pancreatic cancer survival. Mod Pathol. 32(10):1495–507. 10.1038/s41379-019-0291-z 31186528 10.1038/s41379-019-0291-z

[14] Carstens JL, Correa de Sampaio P, Yang D, Barua S, Wang H, Rao A, Allison JP, LeBleu VS, Kalluri R (2017) Spatial computation of intratumoral T cells correlates with survival of patients with pancreatic cancer. Nat Commun 8:15095. 10.1038/ncomms15095 28447602 10.1038/ncomms15095 PMC5414182

[15] Xia Y, Ma J, Yang X, Liu D, Zhu Y, Zhao Y, Fei X, Xu D, Dai J (2024) Identifying the spatial architecture that restricts the proximity of CD8(+) T cells to tumor cells in pancreatic ductal adenocarcinoma. Cancers (Basel) 16(7). 10.3390/cancers16071434 10.3390/cancers16071434 PMC11010991 38611111

[16] Mi H, Sivagnanam S, Betts CB, Liudahl SM, Jaffee EM, Coussens LM, Popel AS (2022) Quantitative spatial profiling of immune populations in pancreatic ductal adenocarcinoma reveals tumor microenvironment heterogeneity and prognostic biomarkers. Cancer Res. 82(23):4359–72. 10.1158/0008-5472.CAN-22-1190 36112643 10.1158/0008-5472.CAN-22-1190 PMC9716253

[17] Mahajan UM, Langhoff E, Goni E, Costello E, Greenhalf W, Halloran C, Ormanns S, Kruger S, Boeck S, Ribback S, Beyer G, Dombroswki F, Weiss FU, Neoptolemos JP, Werner J, D’Haese JG, Bazhin A, Peterhansl J, Pichlmeier S, Buchler MW, Kleeff J, Ganeh P, Sendler M, Palmer DH, Kohlmann T, Rad R, Regel I, Lerch MM, Mayerle J (2018) Immune cell and stromal signature associated with progression-free survival of patients with resected pancreatic ductal adenocarcinoma. Gastroenterology. 155(5):1625-39e2. 10.1053/j.gastro.2018.08.009 30092175 10.1053/j.gastro.2018.08.009

[18] Karamitopoulou E, Wenning AS, Acharjee A, Zlobec I, Aeschbacher P, Perren A, Gloor B (2023) Spatially restricted tumour-associated and host-associated immune drivers correlate with the recurrence sites of pancreatic cancer. Gut. 72(8):1523–33. 10.1136/gutjnl-2022-329371 36792355 10.1136/gutjnl-2022-329371

[19] Karamitopoulou E, Wenning AS, Acharjee A, Aeschbacher P, Marinoni I, Zlobec I, Gloor B, Perren A (2024) Spatial heterogeneity of immune regulators drives dynamic changes in local immune responses, affecting disease outcomes in pancreatic cancer. Clin Cancer Res. 30(18):4215–26. 10.1158/1078-0432.CCR-24-0368 39007872 10.1158/1078-0432.CCR-24-0368

[20] Hanna MG, Ardon O, Reuter VE, Sirintrapun SJ, England C, Klimstra DS, Hameed MR (2022) Integrating digital pathology into clinical practice. Mod Pathol. 35(2):152–64. 10.1038/s41379-021-00929-0 34599281 10.1038/s41379-021-00929-0

[21] Rahman A, Jahangir C, Lynch SM, Alattar N, Aura C, Russell N, Lanigan F, Gallagher WM (2020) Advances in tissue-based imaging: impact on oncology research and clinical practice. Exp Rev Mol Diagn. 20(10):1027–37. 10.1080/14737159.2020.1770599 10.1080/14737159.2020.1770599 32510287

[22] Ung C, Kockx M, Waumans Y (2017) Digital pathology in immuno-oncology – a roadmap for clinical development. Exp Rev Prec Med Drug Dev 2(1):9–19. 10.1080/23808993.2017.1281737

[23] Baddeley A, Rubak E, Turner R (2015) Spatial point patterns: methodology and applications with R. Chapman and Hall/CRC Press, London

[24] Venables WN, Ripley BD (2002) Modern applied statistics with S. Springer, New York

[25] Brooks ME, Kristensen K, van Benthem KJ, Magnusson A, Berg CW, Nielsen A, Skaug HJ, Maechler M, Bolker BM (2017) glmmTMB balances speed and flexibility among packages for zero-inflated generalized linear mixed modeling. The R Journal 9(2):378–400. 10.32614/RJ-2017-066

[26] Therneau TM, Grambsch PM (2000) Modeling survival data: extending the Cox model. Springer, New York

[27] Wickham H. ggplot2 : elegant graphics for data analysis. Cham: Springer International Publishing : Imprint: Springer,; 2016.

[28] Pollini T, Adsay V, Capurso G, Dal Molin M, Esposito I, Hruban R, Luchini C, Maggino L, Matthaei H, Marchegiani G, Scarpa A, Wood LD, Bassi C, Salvia R, Mino-Kenudson M, Maker AV (2022) The tumour immune microenvironment and microbiome of pancreatic intraductal papillary mucinous neoplasms. Lancet Gastroenterol Hepatol. 7(12):1141–50. 10.1016/S2468-1253(22)00235-7 36057265 10.1016/S2468-1253(22)00235-7 PMC9844533

[29] Pea A, Paolino G, Martelli F, Bariani E, Piccoli P, Sereni E, Salvia R, Lawlor RT, Cheng L, Chang D, Scarpa A, Luchini C (2023) Characterization and digital spatial deconvolution of the immune microenvironment of intraductal oncocytic papillary neoplasms (IOPN) of the pancreas. Virchows Arch. 483(2):157–65. 10.1007/s00428-023-03543-4 37086293 10.1007/s00428-023-03543-4 PMC10412653

[30] Opitz FV, Haeberle L, Daum A, Esposito I (2021) Tumor microenvironment in pancreatic intraepithelial neoplasia. Cancers (Basel) 13(24). 10.3390/cancers13246188 10.3390/cancers13246188 PMC8699458 34944807

[31] Versteijne E, van Dam JL, Suker M, Janssen QP, Groothuis K, Akkermans-Vogelaar JM, Besselink MG, Bonsing BA, Buijsen J, Busch OR, Creemers GM, van Dam RM, Eskens F, Festen S, de Groot JWB, Groot Koerkamp B, de Hingh IH, Homs MYV, van Hooft JE, Kerver ED, Luelmo SAC, Neelis KJ, Nuyttens J, Paardekooper G, Patijn GA, van der Sangen MJC, de Vos-Geelen J, Wilmink JW, Zwinderman AH, Punt CJ, van Tienhoven G, van Eijck CHJ, Dutch Pancreatic Cancer G (2022) Neoadjuvant chemoradiotherapy versus upfront surgery for resectable and borderline resectable pancreatic cancer: long-term results of the Dutch randomized PREOPANC trial. J Clin Oncol. 40(11):1220–30. 10.1200/JCO.21.02233 35084987 10.1200/JCO.21.02233

[32] Springfeld C, Ferrone CR, Katz MHG, Philip PA, Hong TS, Hackert T, Buchler MW, Neoptolemos J (2023) Neoadjuvant therapy for pancreatic cancer. Nat Rev Clin Oncol. 20(5):318–37. 10.1038/s41571-023-00746-1 36932224 10.1038/s41571-023-00746-1

