# Supplementary figures and images for: Neoadjuvant therapy-induced remodeling of tumor immune microenvironment in pancreatic ductal adenocarcinoma: a spatial and digital pathology analysis
Source: Virchows Arch. 2025 Feb 27;488(2):277–90. doi: 10.1007/s00428-025-04056-y (PMC12917065; doi:10.1007/s00428-025-04056-y)

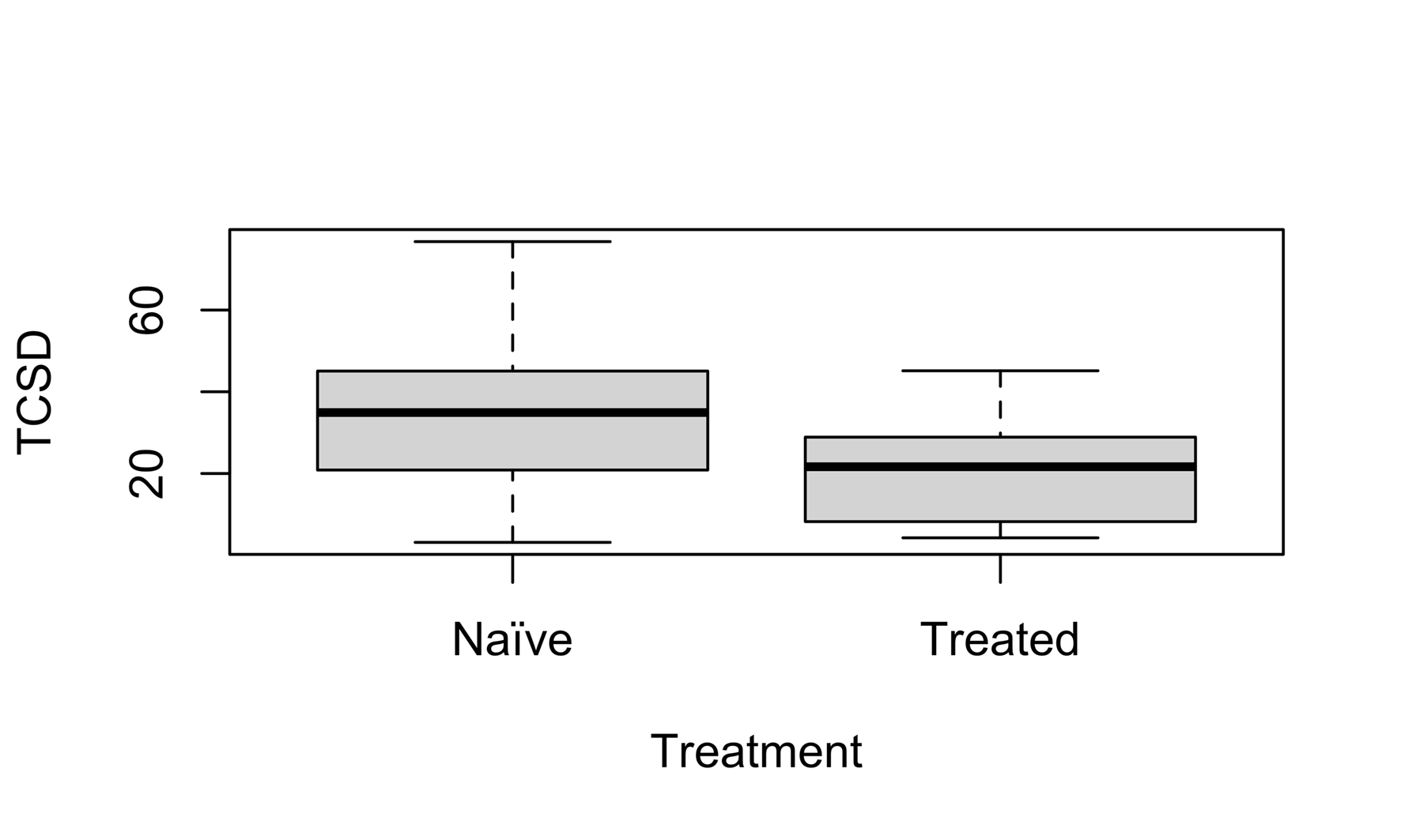

Supplement: Supplementary file 1 — (PNG 39.8 KB) [file 428_2025_4056_Fig7_ESM.png]

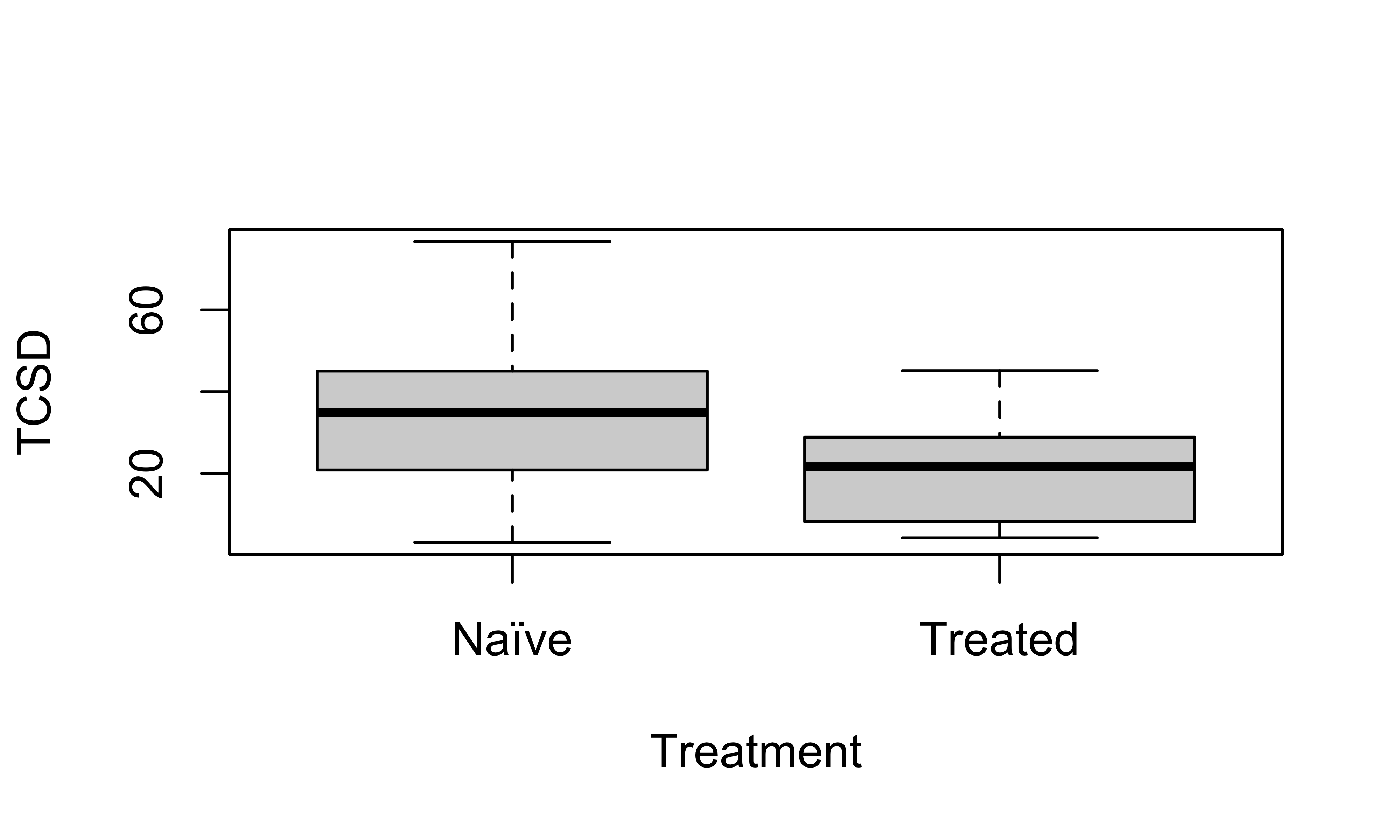

Supplement: Supplementary file 2 — Supplementary file1 (TIFF 37505 KB) [file 428_2025_4056_MOESM1_ESM.tiff]

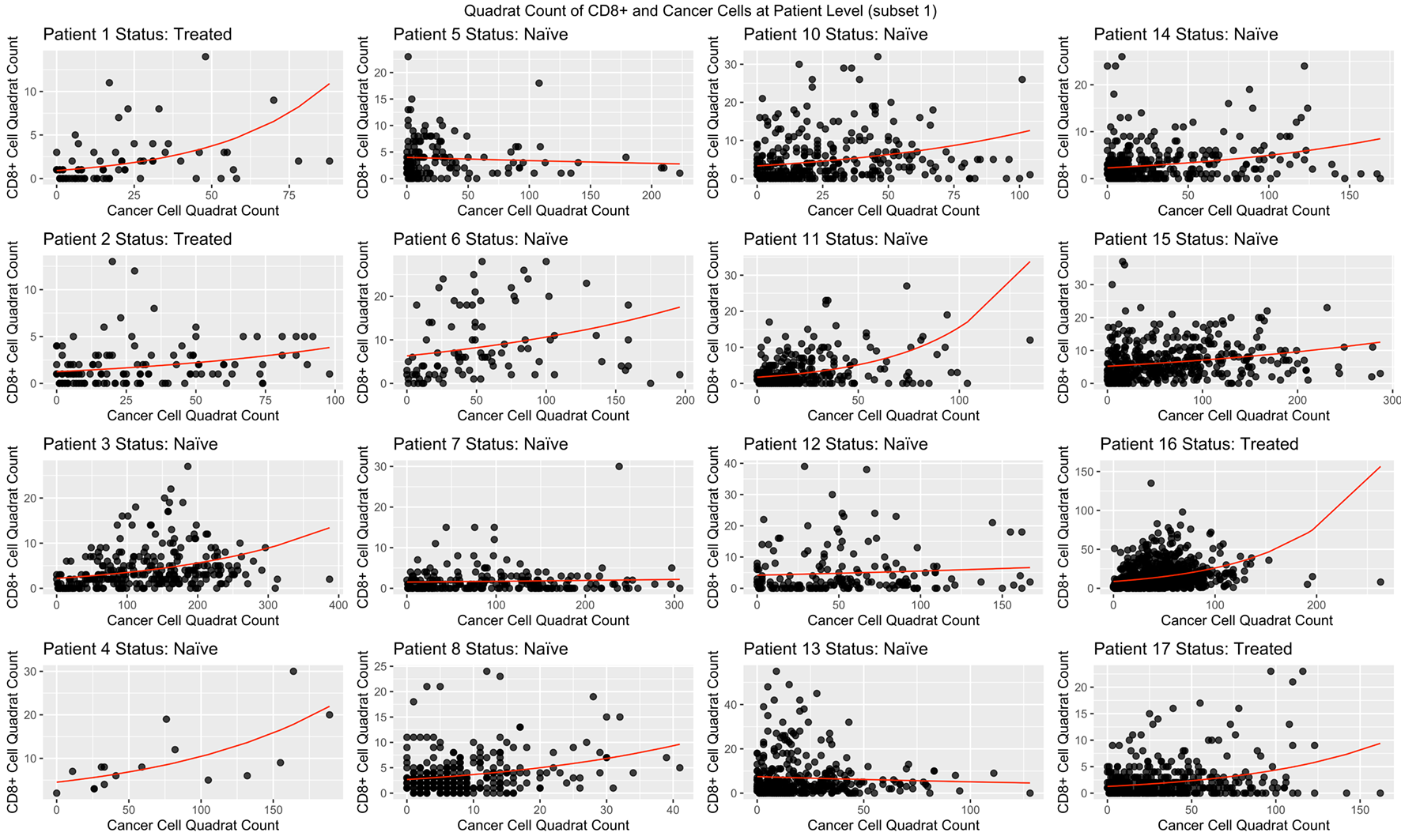

Supplement: Supplementary file 3 — (PNG 534 KB) [file 428_2025_4056_Fig8_ESM.png]

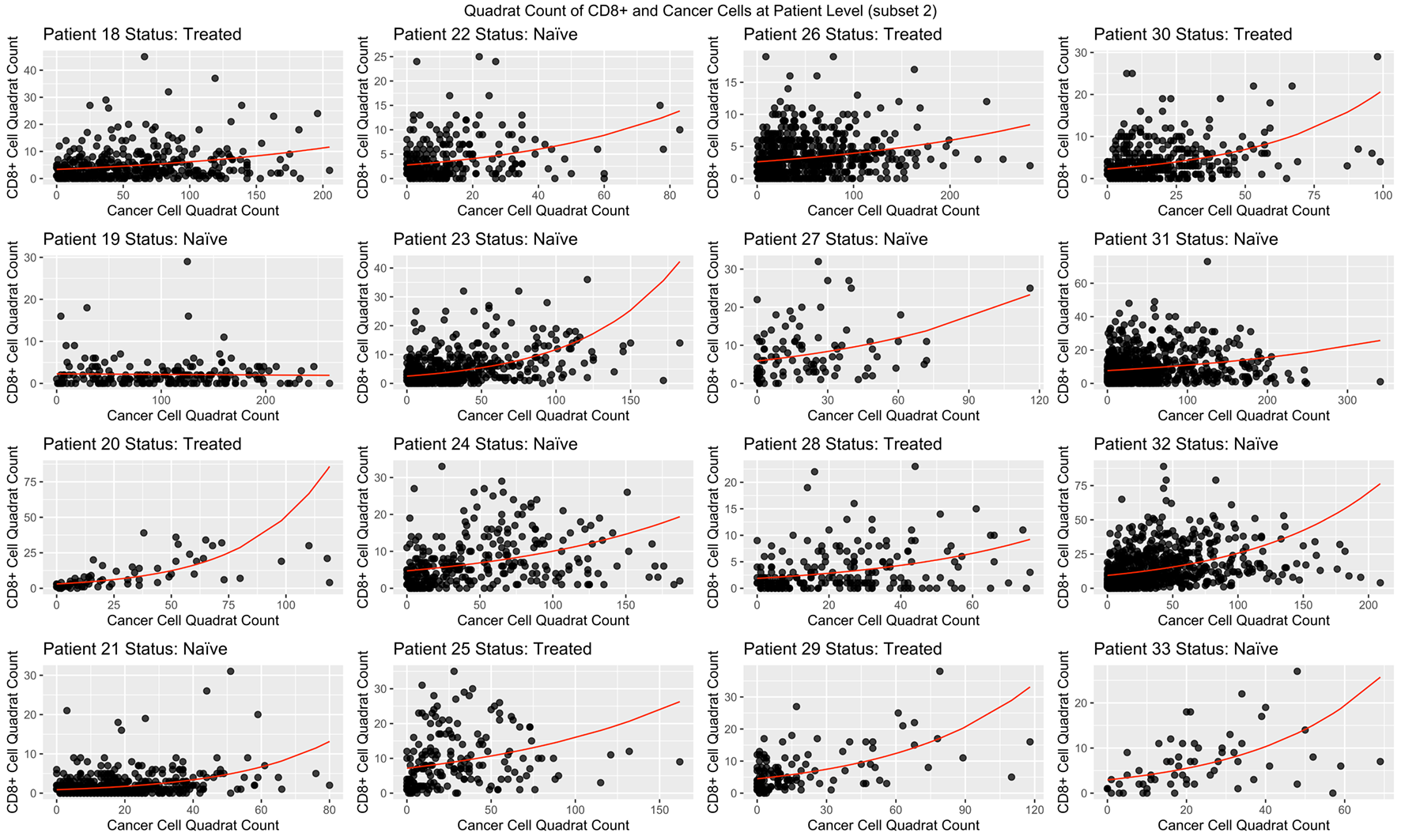

Supplement: Supplementary file 5 — (PNG 562 KB) [file 428_2025_4056_Fig9_ESM.png]

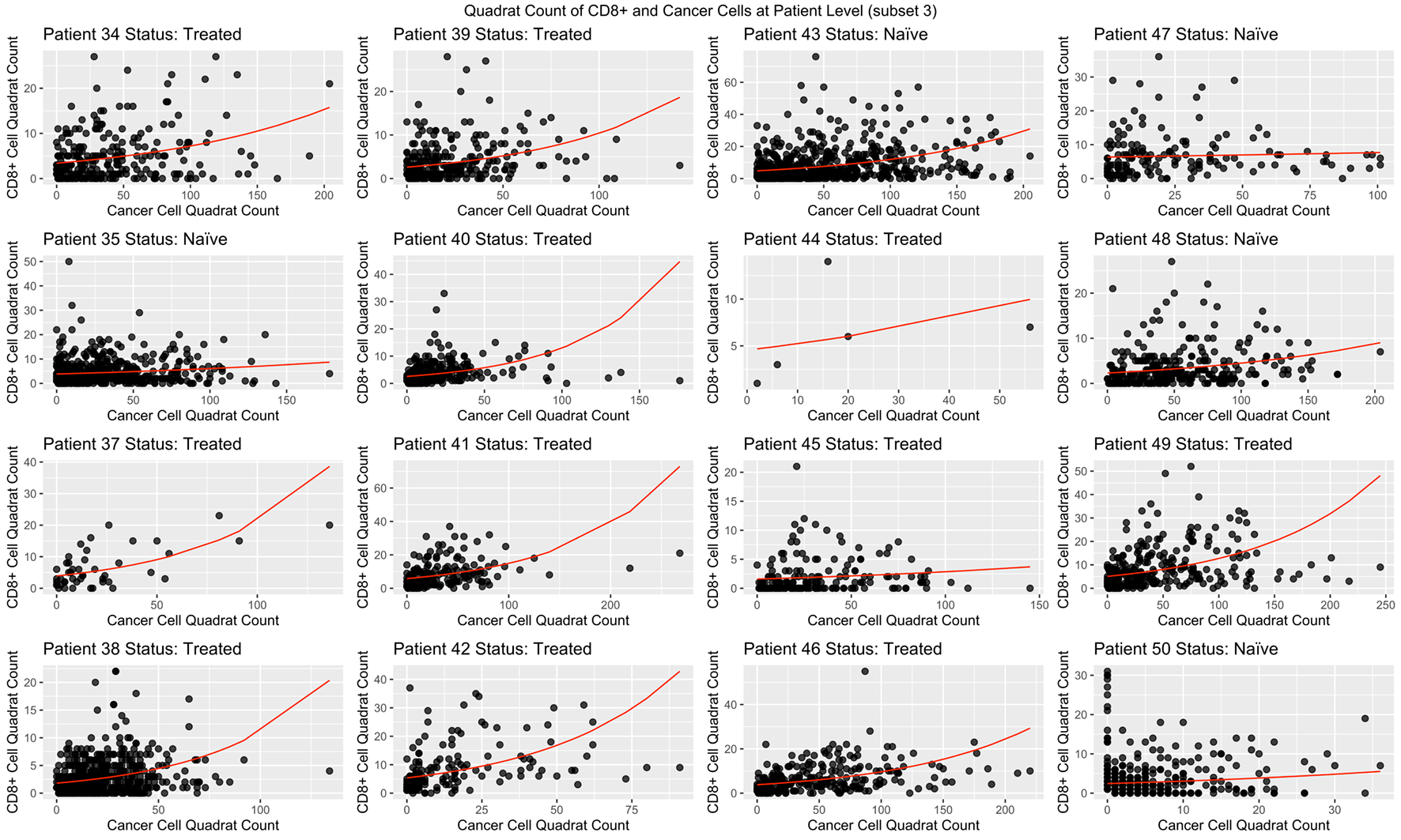

Supplement: Supplementary file 7 — (PNG 519 KB) [file 428_2025_4056_Fig10_ESM.png]

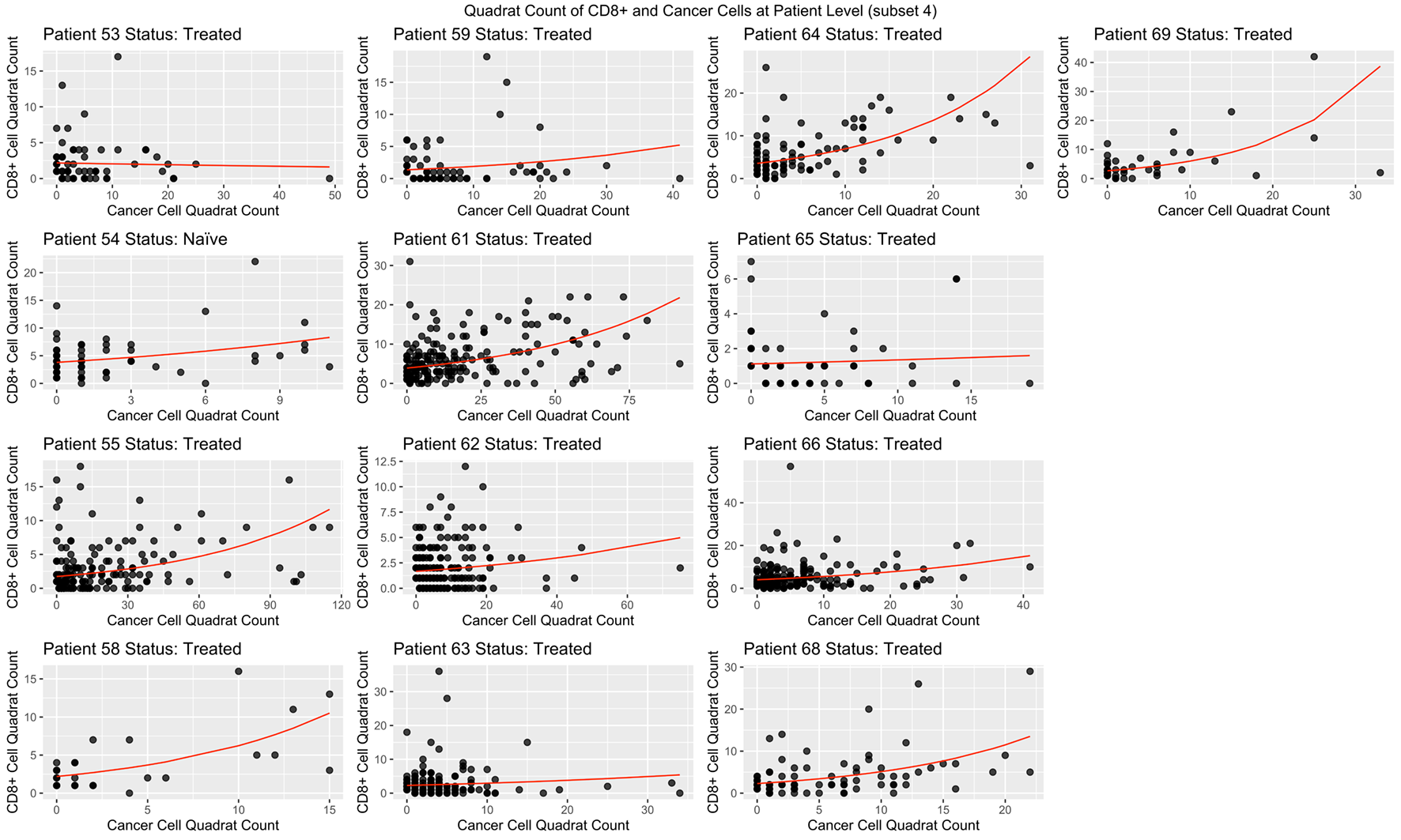

Supplement: Supplementary file 9 — (PNG 376 KB) [file 428_2025_4056_Fig11_ESM.png]
